# Intestinal Inflammation and Altered Gut Microbiota Associated with Inflammatory Bowel Disease Render Mice Susceptible to Clostridioides difficile Colonization and Infection

**DOI:** 10.1128/mBio.02733-20

**Published:** 2021-06-15

**Authors:** Lisa Abernathy-Close, Madeline R. Barron, James M. George, Michael G. Dieterle, Kimberly C. Vendrov, Ingrid L. Bergin, Vincent B. Young

**Affiliations:** a Department of Internal Medicine/Division of Infectious Diseases, University of Michigan, Ann Arbor, Michigan, USA; b Department of Microbiology and Immunology, University of Michigan, Ann Arbor, Michigan, USA; c Department of Biomedical Engineering, College of Engineering, University of Michigan, Ann Arbor, Michigan, USA; d Medical Scientist Training Program, University of Michigan, Ann Arbor, Michigan, USA; e Unit for Laboratory Animal Medicine, University of Michigan, Ann Arbor, Michigan, USA; University of Oklahoma Health Sciences Center

**Keywords:** *Clostridium difficile*, *Helicobacter hepaticus*, animal models, gut inflammation, inflammatory bowel disease, intestinal colonization

## Abstract

Clostridioides difficile is a noteworthy pathogen in patients with inflammatory bowel disease (IBD). Patients with IBD who develop concurrent C. difficile infection (CDI) experience increased morbidity and mortality. IBD is associated with intestinal inflammation and alterations of the gut microbiota, both of which can diminish colonization resistance to C. difficile. Here, we describe the development of a mouse model to explore the role that IBD-induced changes of the gut microbiome play in susceptibility to C. difficile. Helicobacter hepaticus, a normal member of the mouse gut microbiota, triggers pathological inflammation in the distal intestine akin to human IBD in mice that lack intact interleukin 10 (IL-10) signaling. We demonstrate that mice with H. hepaticus-induced IBD were susceptible to C. difficile colonization in the absence of other perturbations, such as antibiotic treatment. Concomitant IBD and CDI were associated with significantly worse disease than observed in animals with colitis alone. Development of IBD resulted in a distinct intestinal microbiota community compared to that of non-IBD controls. Inflammation played a critical role in the susceptibility of animals with IBD to C. difficile colonization, as mice colonized with an isogenic mutant of H. hepaticus that triggers an attenuated intestinal inflammation maintained full colonization resistance. These studies with a novel mouse model of IBD and CDI emphasize the importance of host responses and alterations of the gut microbiota in susceptibility to C. difficile colonization and infection in the setting of IBD.

## INTRODUCTION

Inflammatory bowel diseases (IBDs), including Crohn’s disease and ulcerative colitis, are chronic and progressive conditions characterized by inflammation of the digestive tract. The incidence of Clostridioides difficile infection (CDI) has significantly increased among hospitalized patients with IBD over the past 2 decades (1–3). C. difficile is a spore-forming bacterium that produces enterotoxins that damage the intestinal epithelium (4). C. difficile was initially described as a cause of antibiotic-associated diarrhea (5, 6). Normally, an intact intestinal microbiota provides resistance to C. difficile (7). However, antibiotic exposure can render otherwise healthy individuals susceptible to CDI due to disruption of the microbiota. Although antibiotic use is a well-known risk factor for CDI, other risk factors have been recognized, including immunosuppression and preexisting IBD (8, 9). The gut microbiota plays a critical role in the pathogenesis of IBD (10, 11), and CDI is associated with more severe intestinal microbiota disturbances among patients with IBD (12). In addition, underlying IBD lowers the long-term efficacy of fecal microbiota transplantation (FMT) to treat recurrent CDI, and this is associated with less robust engraftment of donor microbes (13). These results suggest that the pathophysiology of IBD influences gut microbiota composition and CDI outcomes.

The pathogenesis of IBD and CDI have long been studied in animal models (14, 15); however, a robust mouse model of comorbid IBD and CDI in the absence of antibiotic-induced perturbation of the microbiota has yet to be described. Colonization with enteric *Helicobacter* species, including Helicobacter hepaticus, has been shown to trigger colitis in genetically predisposed mice, such as those lacking the regulatory cytokine interleukin 10 (IL-10) (16–18). IL-10^−/−^ mice reared under *Helicobacter*-free, specific-pathogen-free (SPF) conditions develop colitis that resembles human IBD when colonized with H. hepaticus (16, 17, 19), and this intestinal inflammation is associated with alterations in gut microbiota community structures (20). Interestingly, the ability of H. hepaticus to trigger IBD in IL-10^−/−^ mice depends on the presence of an indigenous microbiota, as ex-germfree mice mono-colonized with H. hepaticus do not develop severe colitis (21). Most previously described mouse models of CDI require antibiotic administration to disrupt the intestinal microbiota and render animals susceptible to C. difficile colonization and disease (15, 22) and have revealed that colonization resistance and protection from CDI are mediated by the microbiota and (7, 23) host immune responses (24, 25).

In the present study, we sought to replicate the relationship between IBD and CDI in a mouse model. We wished to develop a system where we could evaluate the specific role of intestinal inflammation and gut microbiota in inducing susceptibility to C. difficile colonization and infection. We utilized a genetic model of murine IBD where intestinal inflammation is triggered by a normal member of the gut microbiota and show that the development of colitis is associated with a loss of colonization resistance to C. difficile.

## RESULTS

### Intestinal inflammation in IL-10*^−/−^* mice colonized with H. hepaticus is associated with altered gut microbiota.

**FIG 1:**
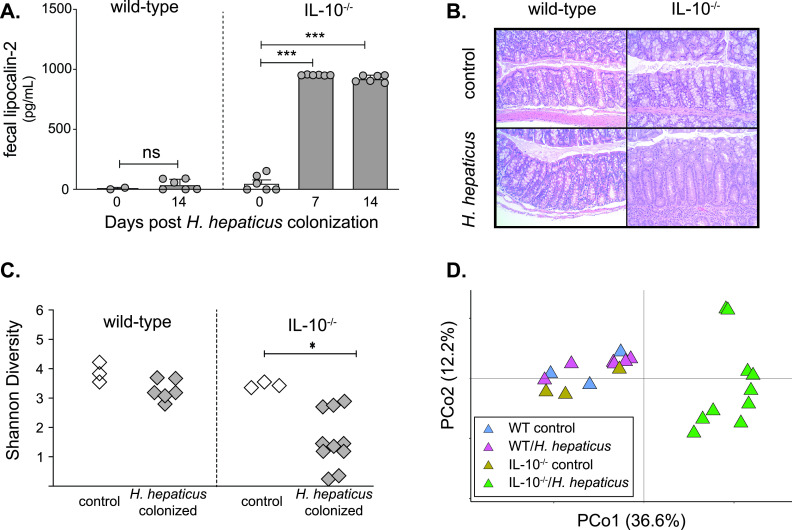
Intestinal inflammation is associated with altered intestinal microbiota in IL-10^−/−^ mice colonized with H. hepaticus. (A) Lipocalin-2 levels in feces from SPF wild-type mice or SPF IL-10^−/−^ mice were measured by ELISA at days 7 and 14 after colonization with H. hepaticus or mock challenged with sterile tryptic soy broth (control). ANOVA and the Tukey test were performed (***, *P* < 0.001). (B) Colonic histologic analysis in SPF wild-type mice or IL-10^−/−^ mice 14 days after colonization with H. hepaticus or mock colonization with sterile broth (control). No histopathologic changes are seen in wild-type animals regardless of H. hepaticus status or in control IL-10^−/−^ mice. The colons of IL-10^−/−^ mice colonized with H. hepaticus had notable epithelial hyperplasia with an acute mucosal and submucosal infiltrate. Representative hematoxylin and eosin images are shown (×50 original magnification). (C) Shannon diversity indexes of the microbial community in the lumens of the proximal colons of wild-type mice or IL-10^−/−^ mice 14 days after H. hepaticus colonization or control. Bacterial community diversity was lower in IL-10^−/−^ mice colonized with H. hepaticus, while no change was seen in wild-type animals following H. hepaticus colonization. A two-tailed unpaired *t* test was performed (*, *P* < 0.05). (D) Principal-coordinate (PCo) analysis plot of Bray-Curtis distances of bacterial communities in the luminal contents collected from the colons of wild-type mice or IL-10^−/−^ mice 14 days after H. hepaticus colonization or from control animals mock challenged with sterile broth.

Wild-type (WT) and IL-10^−/−^ C57BL/6 mice reared under specific-pathogen-free (SPF) conditions received H. hepaticus or sterile broth via oral gavage. Animals were monitored for the development of intestinal inflammation by measurement of the inflammatory marker lipocalin-2 in feces. We confirmed that wild-type mice did not develop signs of intestinal inflammation, regardless of H. hepaticus colonization status, whereas IL-10^−/−^ mice colonized with H. hepaticus did develop colitis. The level of lipocalin-2 was significantly increased in the feces of IL-10^−/−^ mice 7 days after H. hepaticus colonization, and this increase was sustained at 14 days postcolonization (Fig. 1A). Histological examination of colon sections harvested from IL-10^−/−^ mice colonized with H. hepaticus revealed pathology consistent with inflammatory bowel disease, including loss of goblet cells, inflammatory cell infiltration, and crypt elongation 14 days after H. hepaticus colonization, compared to that of WT mice of either genotype mock challenged with sterile tryptic soy broth (Fig. 1B).

We determined whether intestinal inflammation was associated with changes in the gut microbiota. H. hepaticus colonization in WT mice did not significantly impact the diversity of the colonic microbial community (Fig. 1C and D). However, intestinal inflammation in SPF IL-10-deficient animals induced by H. hepaticus colonization was associated with an altered colonic microbial community structure (Fig. 1D). Differences in microbial community structure were driven by treatment (*r*^2^ = 0.193, *P* = 0.0008) and mouse genotype (*r*^2^ = 0.245, *P* = 0.004) (Fig. 1D). Similar changes in the microbiota after the development of colitis were observed in both the cecum and proximal colon (Fig. S1). Taken together, these data demonstrate that the development of IBD in IL-10^−/−^ mice colonized with H. hepaticus is accompanied by alterations in the distal gut microbiota.

10.1128/mBio.02733-20.1 FIG S1 H. hepaticus colonization in SPF IL-10-deficient mice alters the community structure of the distal gut microbiota. (A) The bacterial microbiota alpha diversity, as measured by the Shannon diversity index, is decreased in the cecum and proximal colon of IL-10^−/−^ mice colonized with H. hepaticus. No change was seen in wild-type mice colonized with H. hepaticus or in animals that received sterile culture broth (control). Two-tailed unpaired *t* test (*, *P* < 0.05; **, *P* < 0.01). (B) Principal-coordinate analysis plot of Bray-Curtis distances of bacterial communities in the luminal contents collected from the ceca (circles) and colons (triangles) of SPF WT mice or SPF IL-10^−/−^ mice 14 days after H. hepaticus colonization or mock challenge with sterile broth (control). *Hh*, Helicobacter hepaticus. Download FIG S1, EPS file, 2.1 MB. Copyright © 2021 Abernathy-Close et al. 2021 Abernathy-Close et al. https://creativecommons.org/licenses/by/4.0/ This content is distributed under the terms of the Creative Commons Attribution 4.0 International license.

### The development of IBD in IL-10*^−/−^* animals results in susceptibility to C. difficile colonization.

**FIG 2:**
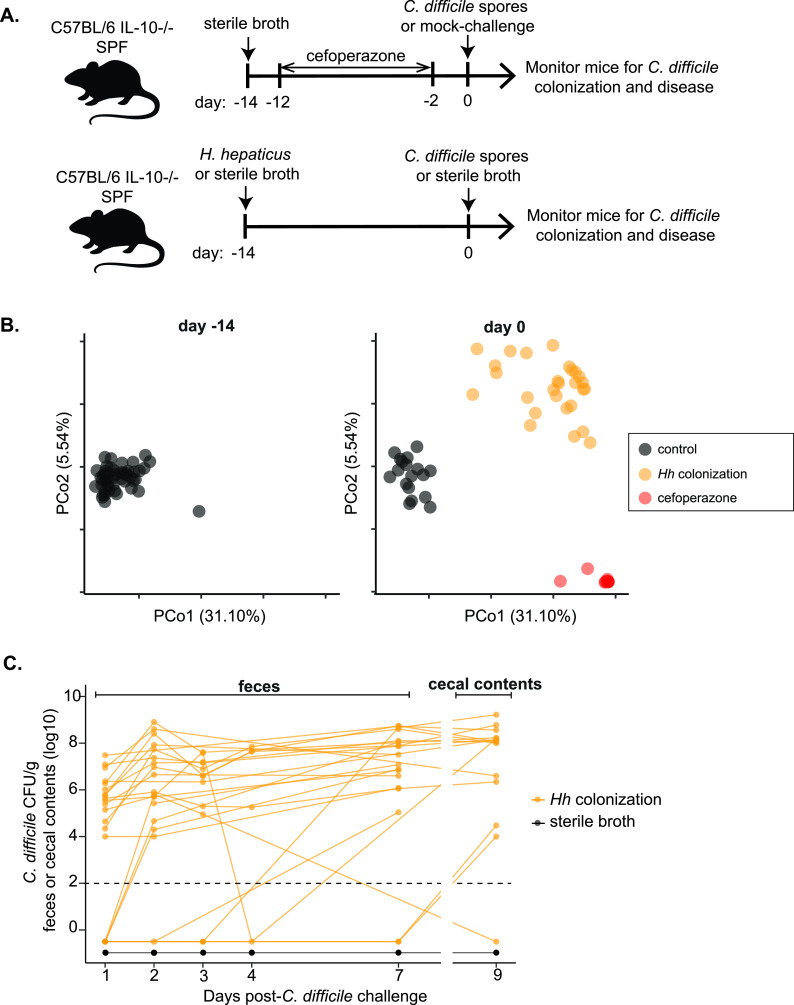
The development of colitis in IL-10^−/−^ animals following colonization with H. hepaticus results in a loss of colonization resistance against C. difficile. (A) Experimental timeline for models of C. difficile infection in IL-10^−/−^ mice. Animals were either treated with the antibiotic cefoperazone to alter their gut microbiota or infected with H. hepaticus to trigger colitis. (B) Principal-coordinate plots of Bray-Curtis distances of bacterial communities in feces collected at baseline (day −14, prior to any experimental treatment) and on the day of C. difficile spore challenge (day 0). Animals that received sterile broth had no change in bacterial community structure at day 0 compared to baseline, while cefoperazone treatment and the development of colitis after H. hepaticus colonization caused marked, but differing, alterations in the microbiota. (C) Colonization dynamics in IL-10^−/−^ mice following C. difficile spore challenge. IL-10^−/−^ mice that had developed colitis after H. hepaticus colonization shed variable amounts of C. difficile in their feces after challenge. Lines show the colonization trajectory of individual mice. All colitic IL-10^−/−^ mice had C. difficile detectable in their feces at some point in the 7 days after challenge, and 8 of 9 mice had C. difficile isolated from cecal contests at the time of necropsy 9 days after spore challenge. No C. difficile was ever recovered from the feces or cecal contents of noncolitic (i.e., noncolonized with H. hepaticus) mice at any point after challenge. The dotted line indicates the limit of detection for C. difficile quantification (10^2^ CFU). Data represent results from 2 independent experiments. A two-tailed unpaired *t* test was performed (*P* < 0.01).

We explored whether mice with intestinal inflammation due to IBD are susceptible to CDI. SPF IL-10^−/−^ mice were colonized with H. hepaticus to trigger IBD prior to challenge with spores of C. difficile strain VPI 10463 (Fig. 2A). As a control for the development of CDI in IL-10^−/−^ animals, we utilized our standard CDI model with antibiotic pretreatment, administering the broad-spectrum antibiotic cefoperazone for 10 days, followed by C. difficile spore challenge (22). Both groups of animals were monitored for C. difficile colonization and signs of clinical disease. As noted above, intestinal inflammation triggered by H. hepaticus colonization was associated with a fecal microbiota structurally distinct from that of noncolonized controls. Furthermore, animals treated with cefoperazone had a fecal microbiota at the time of C. difficile challenge that was different from those of the other two experimental groups (Fig. 2B).

IL-10^−/−^ mice challenged with C. difficile spores in the absence of H. hepaticus-triggered colitis or antibiotic pretreatment were resistant to C. difficile colonization (Fig. 2C). As we have observed previously in wild-type animals, cefoperazone-treated IL-10^−/−^ mice had high levels of C. difficile colonization 1 day after the spore challenge (Fig. S2). Interestingly, 68% of IL-10^−/−^ animals with H. hepaticus-triggered colitis shed C. difficile in their feces 1 day after challenge (Fig. 2C). At 7 days after spore challenge, 89% of mice with IBD shed C. difficile (Fig. 2C). As opposed to what we had observed in antibiotic-treated mice, there was temporal variability in the shedding of C. difficile in the feces of mice with IBD. All mice with IBD had a detectable level of C. difficile colonization at some point during the course of monitoring post-spore challenge, but variation in the levels was seen over the 9 days of the experiment (Fig. 2C).

10.1128/mBio.02733-20.2 FIG S2 IL-10^−/−^ mice pretreated with the antibiotic cefoperazone are colonized with C. difficile and develop severe colitis. (A) Levels of colonization with C. difficile following antibiotic administration are more consistent than in animals with colitis due to H. hepaticus colonization. No colonization was observed in control animals that did not receive antibiotics and were not colonized with H. hepaticus. (B) IL-10^−/−^ animals treated with antibiotics and then challenged with C. difficile spores experience rapid weight loss. (C) IL-10^−/−^ animals treated with antibiotics and then challenged with C. difficile spores experience developed signs consistent with severe clinical infection. (D and E) Cecal (D) and colonic (E) histopathologic scores in animals challenged with C. difficile spores following antibiotic administration, compared to those for control animals and animals that developed colitis after H. hepaticus colonization, with and without subsequent C. difficile challenge. Hh, Helicobacter hepaticus; Cd, Clostridioides difficile; cef, cefoperazone. Download FIG S2, EPS file, 2.8 MB. Copyright © 2021 Abernathy-Close et al. 2021 Abernathy-Close et al. https://creativecommons.org/licenses/by/4.0/ This content is distributed under the terms of the Creative Commons Attribution 4.0 International license.

**FIG 3:**
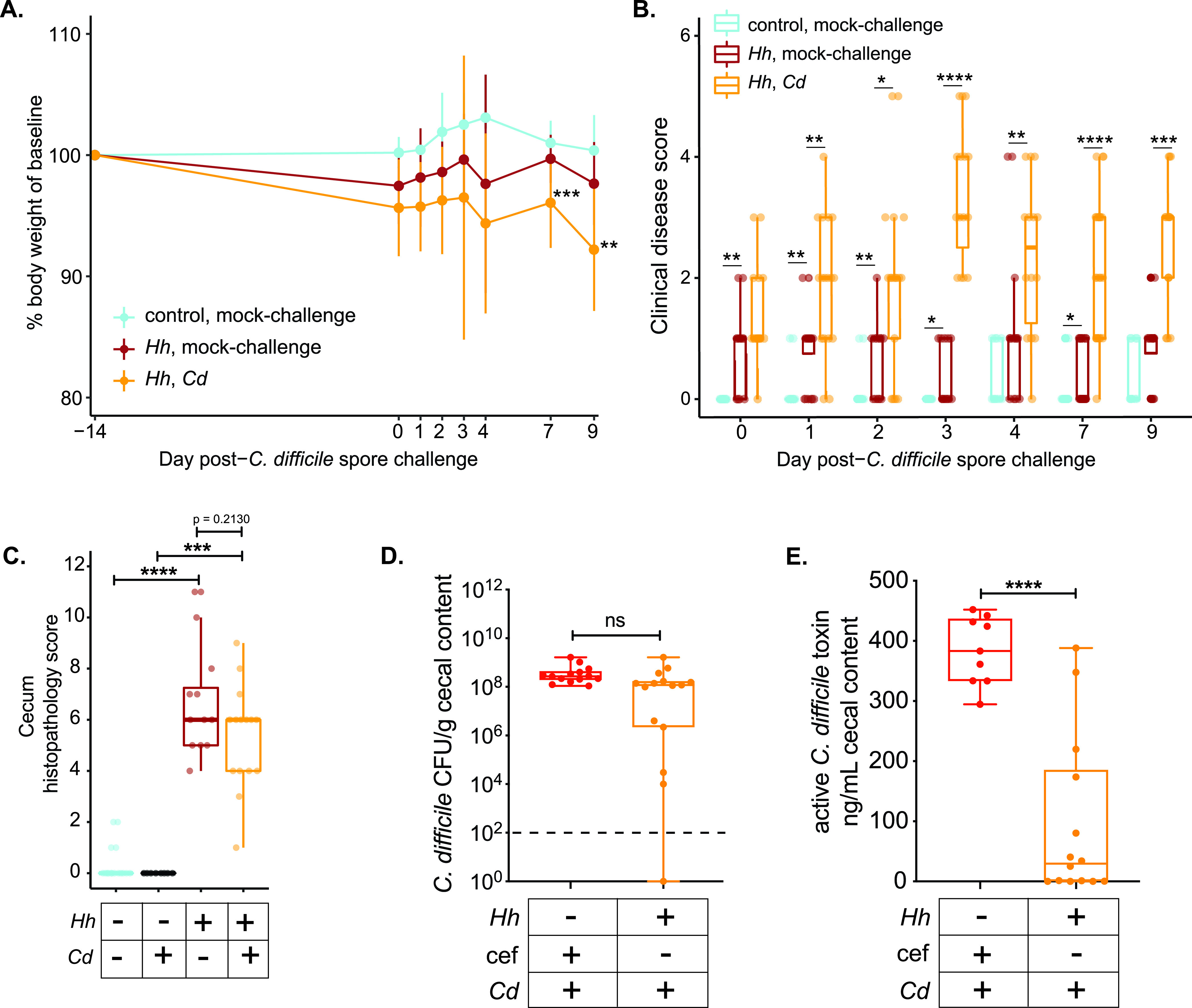
Clinical disease, histopathology, C. difficile colonization, and C. difficile toxin production in IL-10^−/−^ mice with and without colitis following C. difficile spore challenge. IL-10^−/−^ mice were colonized with H. hepaticus (*Hh*) or mock colonized with sterile broth (control). Fourteen days later, both groups of animals received either C. difficile spores (*Cd*) or sterile saline via oral gavage (mock-challenge). (A) Mice that had colitis triggered by H. hepaticus colonization and were subsequently challenged with C. difficile spores (*Hh*, *Cd*) had increased weight loss compared to the weights of animals that developed H. hepaticus-triggered colitis alone (*Hh*, mock-challenge) or IL-10^−/−^ animals that received neither H. hepaticus nor C. difficile (control, mock-challenge). (B) Clinical disease scores. IL-10^−/−^ animals with H. hepaticus-triggered colitis alone (*Hh*, mock-challenge) had higher clinical scores than control animals without colitis (control, mock-challenge). Clinical disease severity was even greater in animals with H. hepaticus-triggered colitis and superimposed C. difficile infection (*Hh*, *Cd*). (C) Cecal histopathology (composite score of edema, epithelial damage, and inflammation) was observed in IL-10^−/−^ animals colonized with H. hepaticus. Superimposed C. difficile colonization in colitic animals did not result in worse histopathology than in mice with colitis alone. (D) C. difficile colonization levels (CFU per gram of cecal contents) in colitic animals challenged with C. difficile spores were not different from those of animals in which colonization resistance was abrogated by administration of the antibiotic cefoperazone (cef). (E) Despite similar cecal burdens of C. difficile in cefoperazone-treated animals and colitic animals challenged with C. difficile spores, greater C. difficile toxin activity was seen in the ceca of animals that had been treated with cefoperazone. Data represent results from 2 to 3 independent experiments and were analyzed by the *t* test (D and E), ANOVA with the *post hoc* Tukey test (A), or the Kruskal-Wallis test (B and C) (*, *P* > 0.05; **, *P* > 0.01; ***, *P* > 0.001; ****, *P* > 0.0001; ns, not statistically significant).

The clinical course of IL-10^−/−^ animals that were challenged with C. difficile after pretreatment with cefoperazone followed what we had reported for wild-type animals (22, 26). At the time of necropsy, animals had gross colitis. Histopathologic analysis revealed severe colitis with edema, epithelial damage, and a marked neutrophilic infiltrate (Fig. S2). Animals with concurrent IBD and CDI lost significantly more weight than mice with IBD alone at day 7 and day 9 after C. difficile spore challenge (Fig. 3A). Overall, IL-10^−/−^ animals with H. hepaticus-triggered colitis and infected with C. difficile had higher clinical disease scores than mice with IBD alone (Fig. 3B). These results suggest that IBD superimposed with CDI results in more severe clinical disease than IBD alone.

Nine days after challenge with C. difficile spores, the IL-10^−/−^ animals were euthanized and tissue was collected for histopathologic analysis. The ceca and colons of mice were examined by a veterinary pathologist and scored for edema, inflammatory infiltrate, and epithelial damage (22). While clinical disease was greater in animals with comorbid CDI and IBD, the histopathology scores were not different between mice with IBD alone and mice with comorbid CDI (Fig. 3C). As the pathogenesis of C. difficile infection is due to the production of the toxins TcdA and TcdB (27), we measured the production of toxin in the ceca of animals at the time of necropsy and quantified the C. difficile burden in the cecal contents of animals with IBD and superimposed CDI. As a group, the level of C. difficile colonization in animals with CDI and IBD was not significantly different from that of animals that were rendered susceptible by antibiotic administration (Fig. 3D). Despite these similar levels of colonization, mice with CDI following cefoperazone treatment had significantly more active C. difficile toxin in their cecal contents than mice with comorbid IBD and CDI (Fig. 3E). Several mice with IBD did not have detectable C. difficile toxin activity, despite being colonized with C. difficile.

### Inflammation following H. hepaticus colonization is necessary to render IL-10-deficient mice susceptible to C. difficile colonization.

**FIG 4:**
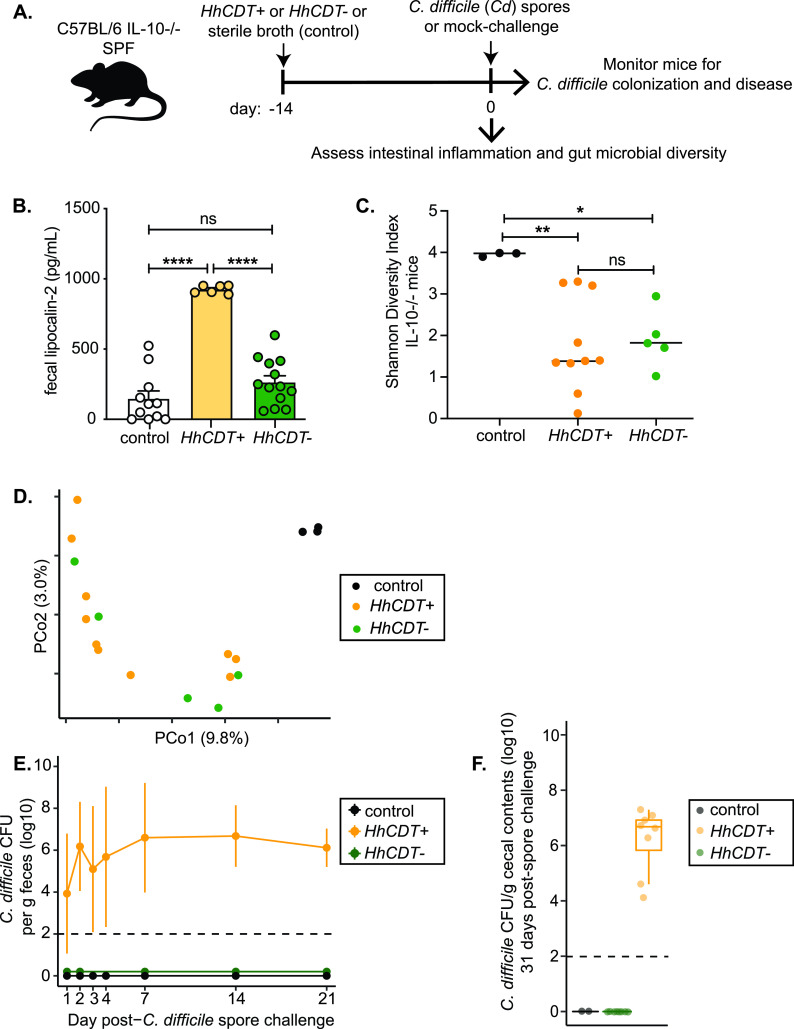
Inflammation in the setting of H. hepaticus colonization of IL-10^−/−^ mice is required to overcome colonization resistance to C. difficile. (A) Experimental design. IL-10^−/−^ animals were colonized with either wild-type H. hepaticus (*HhCDT^+^*) or an isogenic mutant of H. hepaticus that no longer produced the immunoregulatory genotoxin cytolethal distending toxin (*HhCDT^−^*). Mice colonized with either of the H. hepaticus strains were challenged with spores from C. difficile (*Cd*) strain VPI 10463 and monitored for up to 31 days for C. difficile colonization and clinical severity of disease. (B) Lipocalin-2 levels in feces from IL-10^−/−^ mice measured by ELISA at day 14 postcolonization with wild-type H. hepaticus (*HhCDT*^+^) or CDT-deficient H. hepaticus (*HhCDT^−^*). Compared to animals that received only sterile tryptic soy broth (control), animals colonized with wild-type H. hepaticus developed colitis, whereas animals colonized with the CDT-deficient isogenic H. hepaticus mutant did not develop significant intestinal inflammation. (C) Shannon diversity indexes plotted with the group mean. (D) Principal-coordinate analysis plot of Bray-Curtis distances of bacterial communities in the luminal contents collected from the ceca of IL-10^−/−^ mice 14 days after colonization with either *HhCDT*^+^ or *HhCDT*^+^ or after receiving sterile broth (control). Similar changes to the microbiota are seen in animals that were colonized with either strain of H. hepaticus. (E) Shedding of C. difficile in feces collected from mice over time. (F) Cecal contents at day 31 following C. difficile spore challenge were plated anaerobically on selective agar plates to quantify C. difficile burdens (plotted as means ± standard deviations). Only animals that were colonized with wild-type H. hepaticus were colonized with C. difficile.

Our data demonstrate an association between H. hepaticus colonization, the development of gut inflammation, and microbiota changes with susceptibility to C. difficile infection. In an effort to disentangle these potential factors leading to the loss of colonization resistance against C. difficile, we utilized an isogenic mutant of H. hepaticus that is unable to produce cytolethal distending toxin (CDT), a bacterial genotoxin known to modulate immune responses (28). We previously demonstrated that IL-10^−/−^ mice colonized with an H. hepaticus strain deficient in CDT production (*HhCDT^−^*) develop significantly attenuated colitis despite colonization at the same levels as for wild-type H. hepaticus (*HhCDT^+^*) (29, 30). We used this isogenic mutant to further explore the contribution of H. hepaticus colonization and the development of colitis to C. difficile susceptibility in IL-10^−/−^ animals. IL-10-deficient mice were colonized with either the wild-type strain (*HhCDT*^+^) or the *HhCDT*^−^ mutant and compared to controls that received sterile broth via oral gavage. Fourteen days later, all three groups of mice were challenged with C. difficile strain VPI 10463 spores and monitored for C. difficile colonization and disease (Fig. 4A). Mice colonized with wild-type H. hepaticus had significantly higher levels of fecal lipocalin-2 (Fig. 4B) than those colonized with the CDT-deficient mutant and a significantly higher degree of histopathologic intestinal inflammation (Fig. S3).

10.1128/mBio.02733-20.3 FIG S3 CDT-deficient H. hepaticus does not trigger IBD in SPF IL-10-deficient mice. Histopathological damage in ceca and colons collected from SPF IL-10-deficient mice colonized with *HhCDT^+^* or *HhCDT^–^* 31 days after C. difficile spore challenge. Epithelial destruction, immune cell infiltration, and edema were scored on a 4-point scale for each category, and the sum of these scores determined the histological score in each tissue. Data were analyzed using ANOVA with Tukey’s *post hoc* test (*, *P* > 0.05; **, *P* < 0.01; ns, not statistically significant). Download FIG S3, EPS file, 2.7 MB. Copyright © 2021 Abernathy-Close et al. 2021 Abernathy-Close et al. https://creativecommons.org/licenses/by/4.0/ This content is distributed under the terms of the Creative Commons Attribution 4.0 International license.

Colonization of IL-10^−/−^ mice with either *HhCDT^+^* or *HhCDT^−^* was associated with lower microbial diversity in the cecum than in mice that received sterile broth (Fig. 4C). Similarly, examination of the cecal microbial community structure demonstrated that animals colonized with either strain of H. hepaticus were distinct from animals that received sterile broth (Fig. 4D; Fig. S4). The colonization treatment conditions explained 40.7% of the variation in microbial community structure (*P* = 0.0002) (Fig. 4D). Colonization with either *HhCDT^+^* or *HhCDT^−^* was associated with an expansion of *Enterobacteriaceae* and *Lactobacillaceae* and a loss in *Lachnospiraceae* (Fig. S1).

10.1128/mBio.02733-20.4 FIG S4 The inability of H. hepaticus to express CDT does not alter the impact on distal gut microbiota diversity in SPF IL-10-deficient mice. Bray-Curtis distances of bacterial communities in the cecal and colon luminal contents of SPF IL-10-deficient mice 14 days postcolonization with either wild-type H. hepaticus (*HhCDT^+^*) or CDT-deficient H. hepaticus (*HhCDT^−^*), relative to those of mice challenged with tryptic soy broth (control mice). Groups were analyzed by the Mann-Whitney test. Download FIG S4, EPS file, 1.6 MB. Copyright © 2021 Abernathy-Close et al. 2021 Abernathy-Close et al. https://creativecommons.org/licenses/by/4.0/ This content is distributed under the terms of the Creative Commons Attribution 4.0 International license.

Despite similar changes to the gut microbiota in animals colonized with *HhCDT^+^* and *HhCDT^−^*, challenge with C. difficile spores demonstrated differential effects of these two H. hepaticus strains on colonization resistance. C. difficile was not detectable in the feces of IL-10^−/−^ mice colonized with *HhCDT^−^* at any time point following C. difficile spore challenge (Fig. 4E). At the time of necropsy, 31 days after challenge with C. difficile spores, we confirmed this finding in cecal contents and found no detectable C. difficile colonization in mice colonized with *HhCDT^−^*, while animals initially colonized with *HhCDT^+^* had high levels of C. difficile in their cecal contents (Fig. 4F). These data suggest that changes in the microbiota following colonization of IL-10^−/−^ animals with H. hepaticus is not sufficient to lower colonization resistance against C. difficile in the absence of a threshold degree of inflammation.

## DISCUSSION

The clinical intersection between inflammatory bowel disease and C. difficile infection has long been recognized (2). Patients with underlying IBD have an increased incidence of CDI, and this is associated with a worse clinical course (31). Despite our recognition of this relationship between the two conditions, the mechanisms that underlie this confluence between IBD and CDI are not well defined. The indigenous microbiota is one obvious link between these diseases, as alterations in the gut microbiota are thought to play a key role in the pathogenesis of IBD (10, 32). Additionally, an altered microbiota structure and function underly the loss of colonization resistance to C. difficile (33). However, in patients with IBD, the presence of an altered microbiota can be due to a number of factors. It may be related to changes in the microbiota that predispose to IBD, the treatment of IBD with antibiotics or biologics, or the effect of chronic inflammation on the host and the indigenous bacteria. Determining which of these lead to the susceptibility to CDI seen in patients with IBD would be important to help guide rational prevention and treatment strategies.

Clinically, the poor outcomes seen in patients with IBD who have CDI may reflect the direct effect that infection with toxin-producing C. difficile has on the altered epithelium/immune system present in patients with IBD. While this is a straightforward hypothesis, it is interesting to note that a recent retrospective study suggests that patients with more severe IBD are more prone to CDI, and thus, the observed relationship may simply reflect the baseline severity of patients with IBD rather than the C. difficile infection increasing the severity of disease (34). Disentangling the complex relationship between IBD and CDI is challenging due to the lack of appropriate animal models to model this interaction. Recently, reports have investigated how gut inflammation induced by dextran sodium sulfate (DSS) administration in mice influenced subsequent C. difficile infection. Zhou et al. demonstrated that mice with concurrent DSS-induced colitis had a worse clinical outcome when infected with C. difficile (35). Saleh et al. subsequently demonstrated more severe clinical disease due to CDI even when the animals were challenged with C. difficile 3 weeks after cessation of DSS treatment, at a time when the acute colitis due to DSS administration had resolved (36). In both of these studies, susceptibility to C. difficile colonization and increased disease manifestations required the administration of antibiotics before challenge with spores of C. difficile. Zhou et al. did note that about 40% of animals that received DSS without antibiotic treatment could be colonized by C. difficile without antibiotic administration, but in this case, worsened histopathologic colitis was not observed in animals that harbored C. difficile (35). We previously showed that DSS administration alters the fecal microbiota prior to development of severe histopathologic disease (37), and it is possible that this alteration of the microbiota leads to a loss of colonization resistance.

In the current study, we describe a novel murine system that can be used to study the intersection between IBD and CDI in a model that does not require antibiotic administration to render animals susceptible to C. difficile colonization. Furthermore, the model of IBD that we employ is characterized by the development of gut inflammation in a susceptible host that exhibits a dysregulated immune response to the normal gut microbiota. In our SPF colony of IL-10^−/−^ mice, colonization with H. hepaticus rapidly triggers the development of typhlocolitis (20, 29), while no colitis is seen in wild-type animals carrying H. hepaticus. H. hepaticus and other enteric *Helicobacter* species are found as naturally occurring members of the gut microbiota of wild rodents (38–40). Recent studies have demonstrated that inbred laboratory mice carrying microbiota derived from wild mice, which includes *Helicobacter* species, exhibited immune responses that more closely reflected that seen in humans (41, 42). Therefore, the model described here recapitulates the complex relationships between the host, the indigenous microbiota, and the pathogen seen in patients with IBD who are at risk for the development of C. difficile infection.

Our results demonstrate that loss of colonization resistance against C. difficile is not due solely to the changes in microbiota structure that follow colonization with H. hepaticus. While we demonstrate that H. hepaticus colonization of IL-10^−/−^ mice results in an altered cecal and colonic microbiota, the development of inflammation is a critical requirement to overcome colonization resistance. Similar changes in the microbiota of the distal gastrointestinal tract were observed in IL-10^−/−^ animals colonized with either wild-type H. hepaticus or an isogenic H. hepaticus mutant that does not express cytolethal distending toxin. However, only animals infected with wild-type H. hepaticus, which developed much more severe baseline intestinal inflammation, were susceptible to colonization with C. difficile. This indicates that in this system, changes in the microbiota itself do not lead to susceptibility to C. difficile. Only when these changes in the microbiota are accompanied by the development of intestinal inflammation is a luminal environment created that is permissible for the establishment of C. difficile colonization.

The relationship between inflammation, microbiota alterations, and pathogen susceptibility is a theme that has emerged as an important factor in the pathogenesis of a number of gastrointestinal bacterial infections (43). Salmonella has been shown to utilize the alternative electron acceptors present in the lumen of the inflamed intestine to provide a growth advantage over the indigenous microbiota (44). Furthermore, intestinal inflammation limits the availability of micronutrients, including transition metals, such as iron and zinc (45). Some bacterial pathogens have evolved systems to deal with this aspect of so-called “nutritional immunity.” Indeed, the levels of zinc within the gastrointestinal tract have been shown to be a key factor in protection against C. difficile (46).

C. difficile has been shown to be metabolically flexible, allowing it to occupy multiple nutrient niches within the gastrointestinal tract (47). One metabolic feature that has been shown to favor the growth of C. difficile is the presence of luminal amino acids, such as proline, which can be used by the pathogen in energy-generating Stickland fermentation reactions (48). Competition for proline is a critical determinant for the success of C. difficile versus other gut microbes (49). Furthermore, amino acid availability has been shown to be increased in patients with diarrhea, including inflammatory diarrhea, affording a permissive environment for C. difficile (50). The triggering of gut inflammation by the activity of C. difficile toxins was recently shown to specifically alter the intestinal environment in a manner that favors the growth and persistence of C. difficile (51). These findings are in concordance with our current finding that the development of inflammation in a murine model of IBD leads to susceptibility to C. difficile colonization.

The system described here will permit detailed studies of the complex interplay between the indigenous microbiota, host inflammatory responses, and pathogen that underlies the clinical relationship observed between C. difficile infection and inflammatory bowel disease. This model (i) will permit mechanistic studies of the interaction between altered host responses and gut microbes that leads to a breakdown in host-microbe homeostasis and (ii) also will serve as a test bed for novel strategies to prevent and treat C. difficile infection in patients with IBD.

## MATERIALS AND METHODS

### Mice.

Male and female C57BL/6 wild-type or IL-10-deficient mice were maintained under specific-pathogen-free (SPF), *Helicobacter*-free conditions. Mice were at least 8 weeks of age at the start of experiments. All mice were from a breeding colony at the University of Michigan that was originally derived from the Jackson Laboratories in 2002. Euthanasia was carried out via CO_2_ inhalation at the conclusion of the experiment. Animal studies were approved by the University of Michigan’s Committee on the Care and Use of Animals, and animal husbandry was performed in an AAALAC-accredited facility.

### Bacterial strains and growth conditions.

H. hepaticus strain 3B1 (ATCC 51488) was obtained from the American Type Culture Collection (Manassas, VA). The isogenic mutant 3B1::Tn*20* has a transposon inserted near the start of *cdtA* and no longer produces cytolethal distending toxin (CDT) (29). Wild-type H. hepaticus 3B1 and 3B1::Tn*20* were grown on tryptic soy agar (TSA) supplemented with 5% sheep blood at 37°C for 3 to 4 days in a microaerobic chamber (1 to 2% oxygen; Coy Laboratories). The isogenic mutant 3B1::Tn*20* is chloramphenicol resistant and was grown on medium supplemented with 20 μg/ml chloramphenicol (Sigma, St. Louis, MO). Spores of C. difficile reference strain VPI 10463 (ATCC 43255) were prepared and used as previously described by Theriot et al. (22). Spores were enumerated by plating them on prereduced taurocholate cycloserine cefoxitin fructose agar (TCCFA), prepared as previously described (26).

### Infection studies.

H. hepaticus suspensions for animal inoculation were prepared by harvesting organisms from culture plates into Trypticase soy broth (TSB). Mice were challenged with 10^8^ CFU of H. hepaticus by oral gavage. H. hepaticus colonization status was confirmed by PCR of the *cdtA* gene (52) on fecal DNA extracted using a DNeasy UltraClean microbial kit (Qiagen) by following the manufacturer’s instructions. TCCFA plates with fecal or cecal samples or spore inoculum were incubated in an anaerobic chamber (Coy Industries) at 37°C for 18 h prior to colony enumeration.

For our previously published model of C. difficile infection following antibiotic administration, mice received 0.5 mg/ml cefoperazone (MP Pharmaceuticals) in sterile distilled drinking water (Gibco) *ad libitum*. The antibiotic-supplemented water was provided for 10 days, followed by 2 days of drinking water without antibiotics (22). Animals were then challenged by oral gavage with 10^3^ to 10^4^ CFU of C. difficile spores suspended in 50 μl of distilled water (Gibco) or mock challenged with water. To develop a model of C. difficile challenge after the development of colitis, 2 weeks after colonization with H. hepaticus or mock colonization with sterile TSB, animals were challenged by oral gavage with 10^3^ to 10^4^ CFU of C. difficile spores suspended in 50 μl of distilled water (Gibco) or mock challenged with water. Over the course of each experiment, mice were regularly weighed, and feces were collected for quantitative culture. Fresh feces were collected from each mouse into a preweighed sterile tube. Immediately following collection, the tubes were reweighed to determine fecal weight and passed into an anaerobic chamber (Coy Laboratories). Each sample was then diluted 10% (wt/vol) with prereduced sterile phosphate-buffered saline (PBS) and serially diluted onto prereduced TCCFA plates. The plates were incubated anaerobically at 37°C, and C. difficile colonies were enumerated after 18 to 24 h of incubation.

### Clinical disease severity, necropsy, and histopathologic scoring.

Mice were monitored daily for clinical signs of disease. Disease scores were averaged based on scoring of the following features for signs of disease: weight loss, activity, posture, coat, diarrhea, and eyes/nose. A 4-point scale was assigned to score each feature, and the sum of these scores determined the clinical disease severity score (53). At the termination of each experiment, animals were euthanized by CO_2_ inhalation. The cecum and colon were harvested and fixed in formalin. Sections were stained with hematoxylin and eosin and scored by a veterinary pathologist (Ingrid L. Bergin) in a blind manner. Histopathologic damage was scored using epithelial destruction, immune cell infiltration, and edema on a 4-point scale for each category, and the sum of these scores determined the histological score (22, 26, 54).

### Quantitative detection of C. difficile toxin in cecal contents.

The levels of functional C. difficile toxin were measured using a real-time cellular analysis (RTCA) assay (55). The RTCA assay was used to detect changes in cell-induced electrical impedance in cultured colorectal cell monolayers in response to cecal contents collected from mice with CDI to determine concentrations of active toxin. Cecal contents collected from mice at the time of euthanasia were weighed and diluted 1:1,000 (wt/vol) with sterile PBS. After homogenization, particulate matter was allowed to settle in the original collection tubes prior to transference of supernatant aliquots to fresh tubes. Cecal content supernatants were then filtered through a sterile 0.22-μm 96-well filter plate, and plates were centrifuged at 5,000 × *g* for 10 min at room temperature. HT-29 cells, a human colorectal adenocarcinoma cell line with epithelial morphology (ATCC HTB-38), were seeded in electrode-lined 96-well plates (E-Plate View 96; ACEA Biosciences) in Dulbecco’s modified Eagle medium (DMEM) and allowed to grow to a confluent monolayer overnight prior to loading of the processed cecal content supernatant. Samples were run in triplicate. Prior to addition of the cecal content supernatant samples to the plates containing HT29 monolayers, an aliquot of each sample, also run in triplicate, was incubated in parallel with antitoxin specific for C. difficile toxins A and B (C. difficile toxin/antitoxin kit T5000; TechLab, Blacksburg, VA) for 40 min at room temperature as a specificity control for the presence of C. difficile toxin A and B in samples. Active C. difficile toxin has cytotoxic effects on HT29 cells, which results in a dose-dependent and time-dependent decrease in cell impedance (CI). A standard curve was generated using wells that received purified C. difficile toxin A (List Biological Labs). CI data following incubation with cecal contents from mice with CDI were acquired and analyzed using the xCELLigence RTCA system and software (ACEA Biosciences, San Diego, CA). A normalized CI was calculated for each sample by normalizing the CI to the last CI measured at the time point prior to the addition of cecal content to the well.

### Fecal lipocalin-2 quantification.

Fresh feces were collected from individual mice and stored at −80°C until processed and assayed by enzyme-linked immunosorbent assay (ELISA). Fecal pellets were weighed and homogenized in PBS with 0.1% Tween 20. The fecal suspension was centrifuged, and lipocalin-2 levels in the supernatant were quantified using the mouse lipocalin-2/NGAL DuoSet ELISA kit (R&D Systems, Minneapolis, MN) according to the manufacturer’s instructions.

### DNA extraction and 16S rRNA gene sequencing.

Cecal and colon luminal contents were separately collected from mice with IBD and without IBD at the time point immediately preceding C. difficile spore challenge. The University of Michigan Microbiome Core extracted total DNA from cecal and colon contents and prepped DNA libraries as previously described (56). The V4 region of the 16S rRNA gene was amplified from each sample using the dual-indexing sequencing strategy as described previously (57). Sequencing was done on the Illumina MiSeq platform using the MiSeq reagent kit V2 (MS-102-2003) to sequence the amplicons (500 total cycles), with modifications found in the Schloss SOP (https://github.com/SchlossLab/MiSeq_WetLab_SOP). The V4 region of the mock community (ZymoBIOMICS Microbial Community DNA Standard; Zymo Research) was also sequenced to supervise sequencing error. Data were analyzed using mothur (v 1.42.3) (58).

### Statistics.

Statistical analysis for continuous variables was performed using the unpaired Student *t* test or one-way analysis of variance (ANOVA), and Tukey’s *post hoc* test was performed using R. A *P* value less than 0.05 was considered statistically significant. Categorical and ordinal variables were analyzed using nonparametric tests as indicated.

### Data availability.

Code and processing information are available in GitHub repository at the following URL: https://github.com/AbernathyClose/AbernathyClose_IbdCdi_mBio_2020.

10.1128/mBio.02733-20.5 FIG S5 H. hepaticus colonization in SPF IL-10-deficient mice alters the relative abundance of the distal gut microbiota. Relative abundances of bacterial families in cecal and colon luminal contents from mice with IBD (*HhCDT^+^*) and without IBD (*HhCDT^−^*) 14 days after H. hepaticus colonization or mock challenge with sterile broth (control). Download FIG S5, EPS file, 2.1 MB. Copyright © 2021 Abernathy-Close et al. 2021 Abernathy-Close et al. https://creativecommons.org/licenses/by/4.0/ This content is distributed under the terms of the Creative Commons Attribution 4.0 International license.
